# Investigating the Performance of Gesture-Based Input for Mid-Air Text Entry in a Virtual Environment: A Comparison of Hand-Up versus Hand-Down Postures

**DOI:** 10.3390/s21051582

**Published:** 2021-02-24

**Authors:** Yahui Wang, Yueyang Wang, Jingzhou Chen, Yincheng Wang, Jie Yang, Ting Jiang, Jibo He

**Affiliations:** 1 Department of Psychology, Tsinghua University, Beijing 100084, China; yhwangmn@163.com (Y.W.); tan9ochen@gmail.com (J.C.); wang-yc18@mails.tsinghua.edu.cn (Y.W.); nemoyj1989@126.com (J.Y.); 2 Faculty of Psychology, Beijing Normal University, Beijing 100875, China; 201828061046@mail.bun.edu.cn (Y.W.); psytingjiang@gmail.com (T.J.); 3 School of Psychology, Northwest Normal University, Lanzhou 730070, China

**Keywords:** word-gesture text entry technology, gesture recognition, virtual reality, typing performance, subjective experience, task workload

## Abstract

Although the interaction technology for virtual reality (VR) systems has evolved significantly over the past years, the text input efficiency in the virtual environment is still an ongoing problem. We deployed a word-gesture text entry technology based on gesture recognition in the virtual environment. This study aimed to investigate the performance of the word-gesture text entry technology with different input postures and VR experiences in the virtual environment. The study revealed that the VR experience (how long or how often using VR) had little effect on input performance. The hand-up posture has a better input performance when using word-gesture text entry technology in a virtual environment. In addition, the study found that the perceived exertion to complete the text input with word-gesture text entry technology was relatively high. Furthermore, the typing accuracy and perceived usability for using the hand-up posture were obviously higher than that for the hand-down posture. The hand-up posture also had less task workload than the hand-down posture. This paper supports that the word-gesture text entry technology with hand-up posture has greater application potential than hand-down posture.

## 1. Introduction

Virtual reality (VR) has made significant progress in the past ten years, with improvements in both the hardware and software sides. The declining prices of VR products transform VR into a new technology of mass consumption that is widely used by game developers, researchers, and ordinary consumers. At the same time, the quality of graphics in VR is getting higher and higher. The realistic graphics and full immersion experience make VR technology more and more popular.

Although the interactive technologies of VR systems have made great progress in recent years, the accuracy of symbol and text input in VR is still an urgent problem to be solved. Compared with other interactive tasks in a virtual environment, text input is a more complex and refined task. Prior researchers have investigated various interaction methods for typing in VR, such as wearable devices [1], VR controllers [2,3], head and gaze devices [4], pens and tablets [5], virtual touchscreens, and augmented reality keyboard [6,7], speech-to-text input device [8], and hands or gestures [9,10].

**Figure 1:**
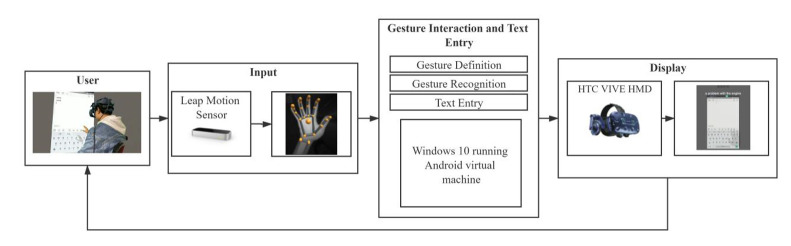
The technical architectural diagram of the gesture-based mid-air text entry technology.

**Figure 2:**
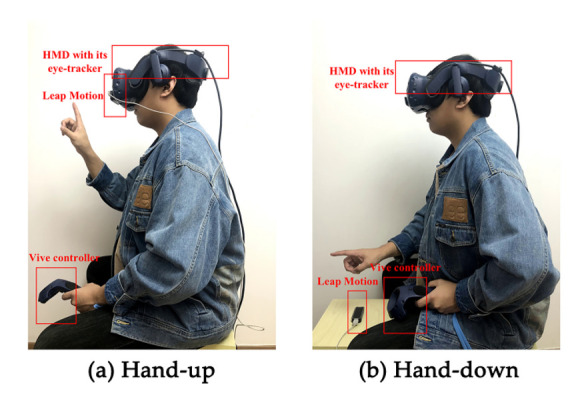
The different input postures. (**a**) Hand-up; (**b**) hand-down.

In this study, we deployed a gesture-based mid-air text entry technology based on hand tracking in the virtual environment. We used Leap Motion for input, and transported hand information to a Windows 10 laptop, then conducted gesture definition and gesture recognition to control a cursor. An Android word-gesture keyboard was run on this laptop, and users controlled the cursor to draw the gesture path in mid-air on the word-gesture keyboard. Users used a continuous gesture path to traverse each letter of the word to be entered, and then the word-gesture keyboard matched the corresponding word according to the gesture path. The desktop image of this laptop was transported to HMD by Unity in real-time, so users can watch the word-gesture keyboard, the cursor, and hand model through HMD. Figure 1 is a technical architectural diagram of this text entry technology. We provided two different interaction hand postures (hand-up vs. hand-down) for this input method. The interaction posture in this study refers to the posture of the dominant hand side of the human body from the elbow joint to the fingertip in the process of text input. The hand-up posture is that the body parts mentioned above are perpendicular to the ground, and the hand-down posture means that they are parallel to the ground, as Figure 2 shows.

The use of gestures to interact has shown promise as a natural way for human beings to interact with digital information in virtual environments [11]. However, even for a medium time aerial gesture, users will feel arm fatigue and discomfort, thus leading to gorilla arm syndrome [12]. Existing studies have proved that the hand-up posture is more likely to lead to fatigue and gorilla arm syndrome than hand-down posture [12]. Thus, in virtual environments, we speculated that the hand-down posture’s text input performance is better than that of the hand-up posture in this study. In addition, users’ familiarity with VR will affect their interaction efficiency, and we inferred that users with VR experience will perform better than those with no VR experience in this study. Furthermore, given the critical influence of input posture on the efficiency of human–computer interaction in VR, we conjectured that the differences of hand posture had greater influence than the VR experience on input performance when using the mid-air gesture-based text entry technology in the virtual environment.

This research aimed to investigate the impact and performance of using different postures to input text by word-gesture technology in the virtual environment and the interaction between different postures (hand-up vs. hand-down) and users with different experiences of VR (with VR experience vs. without VR experience). This study discussed the effects of posture and the interaction between the two independent variables from the dimensions of typing performance, subjective experience, and the task workload to provide references and suggestions for the future research of word-gesture text entry technology based on gesture recognition in the virtual environment.

The remainder of the paper is organized as follows. Section 2 presents related studies on text entry in virtual environments, mid-air entry methods, and word-gesture text entry studies in a virtual environment. The materials and methods we used to collect data and the study setup are presented in Section 3. The results of the research and discussion about the findings are presented in Section 4 and Section 5, followed by the conclusion and future work in Section 6 and Section 7.

## 2. Related Works

### 2.1. Text Entry in Virtual Environments

There have been some researchers who study text entry methods in virtual environments. The controller was considered as an efficient pointing method for text entry [13]. Speicher et al. [14] found the entry rate of Controller Point and Select to be around 15.44 WPM in VR. Using a Swype-like algorithm, Jimenez et al. [15] developed a prototype for text input in VR, combining a novel continuous-motion text input method with hand postures. It reduced the waiting time compared with hover-based programs, but its auto-correct performance was not reliable. Lu et al. [16] compared three hands-free entry methods in VR: BlinkType, NeckType, and DwellType. Results showed that the mean typing speeds for BlinkType, NeckType, and DwellType were 13.47, 11.18, and 11.65 WPM, respectively.

Some researchers focused on the improvement of keyboard design in VR. A punching type keyboard with keys arranged in three dimensions was presented by Yao et al. [17] in VR. The performances of touchscreen keyboards and desktop keyboards in VR were compared by Grubert et al. [18]. They found that the typing performance of novice users on a desktop keyboard is better than that on a touchscreen keyboard. Boletsis and Kongsvik [2] evaluated the drum-like VR keyboard. Their experiment showed this keyboard (typing at 24.61 WPM) competing against the rates of other interfaces such as a head-based interface. Yeo et al. [19] mentioned that the tilt-based gesture keyboard on smartphones could achieve the maximum text entry rate at 32 WPM, which meant it could be used in VR. A hand-or finger-mounted data input device with a QWERTY layout was developed by Kuester et al. [20], but it did not rely on a keyboard and could be used in AR/VR environments. Although the keyboard designs in VR affected the performance of text entry as mentioned in the above papers, the input gesture of users is another important factor impacting the user experience, which is ignored by the related works.

In addition, previous works usually investigated the efficiency of the entry method and reported participants’ subjective satisfaction [21,22], and the performance of the methods in VR is evaluated by various factors, such as typing speed and ease of use [23]. The related papers paid much attention to the typing speed but ignored the user experience of participants. Therefore, in this paper, we focus on the gesture-based input method for mid-air text entry in VR by comparing the typing performance, subjective experience, and task workload when using the hand-up posture and the hand-down posture.

### 2.2. Mid-Air Text Entry Method

Mid-air text entry is used widely in activities such as doing presentations and gaming. Two main forms from the research on the mid-air entry method are the selection-based method and gesture-based method. Shoemaker et al. [24] developed three selection-based methods for mid-air text entry using three layouts: 3D cube, circular, and QWERTY. The experiment showed that QWERTY (18.9 WPM) performed significantly better than the other techniques. Markussen et al. [25] adopted three text-entry methods, H4 MidAir, MultiTap, and QWERTY, to achieve selection-based mid-air text entry. The mean typing speed of the QWERTY-based text-entry method was 13.2 WPM, which was the fastest among the three methods. Some research explored the mid-air text entry in terms of the virtual keyboard layouts and the entry method. Ren and O’Neill [26] and Ren et al. [27] studied the mid-air text entry with QWERTY layout and dual circle layout and found the dual circle layout performed better in ease of use and error tolerance. This result contrasted with the result, which was further verified by Gijs et al. [28], and it indicated that the QWERTY layout with intelligence was better in handheld devices. Using accelerometers, the gestural text-entry system incorporating the virtual keyboard with matrix layout and tri-center layout was designed for mid-air text entry [29]. The experiment result showed that the matrix-based layout was more suitable for novice users.

The freehand mid-air as a promising input method has attracted researchers’ attention. Several studies were related to users’ preference for input gestures. Wobbrock et al. [30] proposed a unified approach to elicit gestures from users with the aim of maximizing and evaluating the guessability of gestures. A study on the guessability of input gestures on a three-meter wide display was conducted by Wittorf and Jakobsen [31]. They found that participants would like to vary gestures for pointing, grabbing, and swiping, and, 55% of the proposed gestures were physical in nature. In the work of Wobbrock et al. [32], the result also showed that 44% of users preferred physical gestures.

The mid-air text entry has also been applied to VR; Adhikary presented an interactive mid-air text entry system in VR and investigated user performance when using one hand or two hands on a split keyboard [10]. Yu et al. [33] compared three input techniques: TapType, DwellType, and GestureType in VR, and improved the gesture-word recognition algorithm, achieving the typing speed of 24.4 WPM for trainees. Some researchers focused on the mid-air gesture elicitation in AR. Piumsomboon et al. [34] extended Wobbrock’s surface taxonomy to AR environments and created a user-defined gesture set in AR. Based on VR and AR maps, Giannopoulos et al. [35] explored a combination of mid-air hand postures with head gestures for head-mounted displays. A gesture elicitation study to explore the applicable interactive gestures for large immersive AR maps was conducted by Austin et al. [36]. The results of gesture sets, such as panning and rotating, could guide the development of gesture interaction and immersive maps. For home devices in smart environments, Vogiatzidakis and Koutsabasis [37] developed a spatial AR prototype to test the mid-air gesture and found the interaction between mid-air and home devices was possible.

Prior research often explored the mid-air entry method concerning the typing efficiency in VR. However, researchers need to pay more attention to what postures and gestures should be adopted by users for text entry in a virtual environment.

### 2.3. Word-Gesture Text Entry

Word-gesture text entry method enabled users to “draw a word” on the input surfaces. In terms of the gesture-based method, Zhai and Kristensson [38] proposed a shorthand writing method, SHARK, which was the first word-gesture text entry method. Their user study showed that participants could learn SHARK at the average speed of 15 words per 45 to 50 min. Later, they improved SHARK in terms of the alternation between the two modes of writing, i.e., tapping and gesturing, and named it SHARK2. It was possible to achieve real-time performance on mobile devices [39].

Markussen et al. [40] designed a keyboard for mid-air entry named Vulture based on touch and word gesture, and its input rate could reach 20.6 WPM. Based on Leap Motion, Zhang et al. [41] designed AirTyping, i.e., a typing solution allowing people to type in mid-air without devices. The results showed that the true positive rate of keystroke detection was 92.2%. Two novel word-gesture input techniques using six degrees of freedom VR controllers (16.4 WPM) and pressure-sensitive touchscreen devices (9.6 WPM) were proposed by Chen et al. [42]. The experiment result showed that the former was better than the latter in usability and task load. A word-gesture typing technique named RotoSwype was designed by Gupta et al. [43], which uses a wearable ring to orient. This typing method could achieve unencumbered, one-handed, and eyes-away typing on any QWERTY keyboard on any device after a long period of practice (five days). The average typing speed was faster than 14 WPM with a near 1% uncorrected error rate. Although RotoSwype with a ring was portable, that is still not completely freehand.

Previous studies paid little attention to the impact of the word-gesture technology on cognition and ergonomics in a virtual environment, few studies have explored the usability of word-gesture text entry with different postures in the virtual environment.

## 3. Materials and Methods

This study assessed and compared the typing performance and user experience of typing in a virtual environment with two different interactive postures (hand-up and hand-down), while two user groups (with or without the experience of using VR devices) used the word-gesture text entry technology. Further, we measured the perceived usability, task workload, perceived exertion, and motion sickness to evaluate the subjective experiences of different input postures.

Leap Motion was set in different positions in different conditions. In the hand-up posture condition, Leap Motion was set on the HMD. In the hand-down posture condition, Leap Motion was set on a platform in front of the participant, which was at the same height as the participant’s seat. It was made to keep and ensure the participant’s hand within the detection range of Leap Motion. The schematic diagram of two input postures and Leap Motion are shown in Figure 2.

### 3.1. Participants

**Figure 3:**
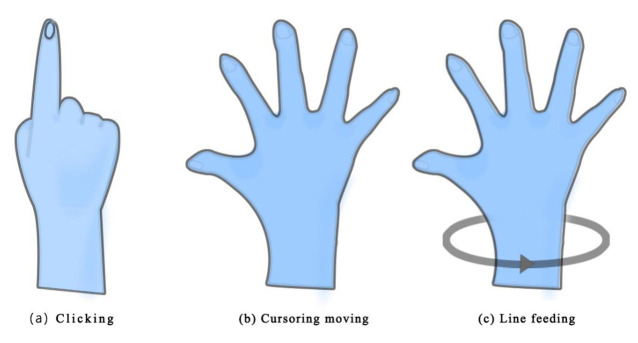
The different interactive gestures. (**a**) Clicking; (**b**) cursor moving; (**c**) line feed.

We recruited 35 participants (18 males and 17 females), aged from 18 to 29 (*M* = 21.17, *SD* = 3.129), to participate in this study. Participants were recruited based on their VR use experience. Participants with the experience of using VR devices were required to have used VR devices more than five times in the past year, and the total use time was more than 1 h. Participants without the experience of using VR devices were required to have no experience in VR. Eighteen participants without VR experience (10 females, eight males) and 17 participants with VR experience (nine males, eight females) were involved; none had experience using the word-gesture text entry technology. All participants typed using the only input rules, as Figure 3 shows. All participants were right-handed. All participants had normal vision or corrected to normal vision and had no hand, arm, shoulder, or neck disease (pain).

### 3.2. Materials

We conducted the study in a virtual reality environment that consisted of a head-mounted virtual reality display (HTC Vive PRO; display: 110° horizontal field of view; resolution: 1440 × 1600 pixels per eye; refresh rate: 90 Hz), two Windows10 laptops (The first one: CPU i7-8750H 2.20 GHz, RAM 16.0 GB, GPU NVIDIA GeForce GTX 1070. The second one: CPU i5-1035G1 1.30 GHz, RAM 16.0 GB), and a Leap Motion Controller (140° × 120° typical field of view; interaction depth between 10 cm to 60 cm preferred, up to 80 cm maximum). The controller’s triggers of HTC Vive PRO can switch to the next phrase during experiments.

The study used a virtual machine running Android 5.5.1 and the leap motion capture program. Additionally, the virtual machine was equipped with the word-gesture text entry application (Google Input Application). The real-time user interface of the Android virtual machine was displayed in a VR HMD through a unity program, and the time delay of the image from Android to VR was less than 30 ms. The leap motion capture program was developed to capture the gesture and position of the user’s right hand. Participants could manipulate the cursor by controlling the coordinates of their right index finger. We have designed a series of interactive gestures for users through the program, which could realize functions such as clicking, cursor moving, and line feeding. The interactive gestures are shown in Figure 3.

When typing by the word-gesture text entry technology, users needed to perform the following. First, use the gesture (b) to move the cursor to the first character of the word that needed to be input. Then use gesture (a) to click the first letter and keep this gesture and move the hand to control the cursor on the screen. Participants need to keep this gesture and control the cursor to pass through all the characters of the word to be input and then resume gesture (b). At this time, the participant completed inputting one word. When the participant wanted a line feed, he needed to turn his palm 180° as gesture (c) and then resume gesture (b) (see Figure 3).

**Figure 4:**
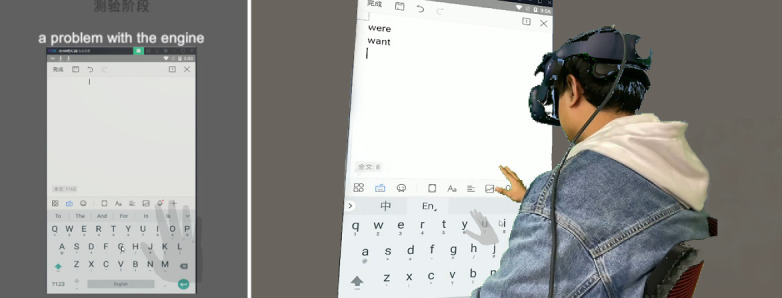
The virtual environment including stimulus, Android virtual machine user interface.

The virtual environment consisted of a 0.62 × 1.06 m^2^. Android user interface, and the stimuli were on it. At 2 cm over the user interface, the phrases that participants need to enter were displayed. Participants were one meter away from the user interface. Participants could see a grey hand model with a transparency of 50% in the virtual environment. This hand model was generated based on the position and gesture of the participant’s hand and could reflect the participant’s real hand movements. The model was captured by Leap Motion and then generated in Unity. Except for these three elements, there was only a grey background around, which allowed participants to focus on the task. Participants could see the cursor on the virtual machine user interface and manipulate the cursor through gestures (see Figure 4).

We randomly sampled a subset of phrases for each participant under each condition from a list of 500 phrases composed by MacKenzie and Soukoreff [44]. It included 15 practice phrases and 10 experimental phrases. The phrases contained lowercase letters only (no number, symbol, punctuation, or uppercase letter), which are 16 to 43 characters in length.

### 3.3. Procedure

First, participants needed to sign the informed consent voluntarily. Before the formal experiment, participants used a Windows10 laptop and the Leap Motion to learn the word-gesture text entry technology in reality for about 1.5 h and followed an about 3-min VR tutorial of learning how to use the VR HMD and controllers. 

Next, they entered the practice phase before the experiment phase. In the practice stage, participants transcribed 15 practice phrases randomly sampled from a list composed by MacKenzie and Soukoreff [44] under each condition. The order of conditions was counterbalanced.

In the experimental phase, the order of conditions was counterbalanced. There were 10 experimental phrases for participants under each condition. It only presented one phrase at a time. Participants could switch to the next phrase by the controller’s trigger when they completed a phrase. The phrase was shown in VR over the Android virtual machine user interface 2 cm. In this phase, participants were required to transcribe each phrase as quickly and accurately as possible, but they were not allowed to use backspace to correct errors. In order to facilitate the calculation of the word accuracy, participants required a line feed when they completed each word rather than each phrase.

Once the participants had completed one condition, they were asked to take off the HMD and fill out subjective questionnaires, and rest for at least 15 min. After all tasks, they were asked to fill out a demographic questionnaire, including age, gender, and experience. Each participant spent about 180 min in total.

### 3.4. Design

A 2 × 2 mixed design was adopted. The within-subject factor was input posture (hand-up vs. hand-down), and the between-subject factor was VR experience (with VR experience vs. without VR experience). Dependent variables included typing performance, subjective experience, and task workload. 

#### 3.4.1. Typing Performance 

We measured the words per minute and the word accuracy rate of 10 phrases to evaluate the typing speed and accuracy. 

Words per minute (WPM) was calculated by the following formula:

WPM = (|T| − 1)/S × 60/5(1)

where |T| is the number of transcribed characters in the experimental phase; S is the number of seconds in the experimental phase. Each word is presumed to be five characters [45].

Word accuracy (WAcc) was calculated by dividing the number of correct words by the number of words in the experimental phase.

The learning effect of typing accuracy was measured by comparing the word accuracy of the practice phase and the experimental phase.

#### 3.4.2. Subjective Experience

We used several subjective feedback questionnaires to assess participants’ subjective experience, as follows:

System Usability Scale (SUS): an industry-standard questionnaire to assess participants’ perceived usability of each input posture. It consists of 10 items, each with five options (Strongly Disagree to Agree). All responses are summarized as an overall result between 0–100 [46]. The higher the score, the better the perceived usability.

User Experience Questionnaire (UEQ): a seven-point scale to assess the user experience of each input posture. It consists of 26 items covering six factors (Attractiveness, Perspicuity, Efficiency, Dependability, Stimulation, and Novelty). They are grouped into three qualities, attractiveness, pragmatic quality, and hedonic quality, and the score of each is between −3 and 3 (the most negative answer to the most positive answer) [47].

Virtual Reality Sickness Questionnaire (VRSQ): a four-point scale to assess motion sickness in the virtual environment. It consists of nine items and covers two dimensions of VR motion sickness: oculomotor and disorientation. We calculated a total score between 0 and 100 of VR motion sickness. The lower the score, the less motion sickness [48].

Borg CR10 Scale: a general scale to measure perceived exertion, and the range is from 0 to 10. The lower the score the less perceived exertion [49].

#### 3.4.3. Task Workload

Task workload metric was measured by NASA TLX. NASA TLX is an industry-standard questionnaire to assess subjective workload, and it covers six facets (mental, physical, time, effort, performance, and frustration). The higher the score, the more workload or the worse perceived performance [50].

## 4. Results

All dependent measures were analyzed using a 2 × 2 mixed-design ANOVA, while the learning effect was analyzed using a paired T-test.

### 4.1. Typing Performance

#### 4.1.1. Words Per Minute (WPM)

**Figure 5:**
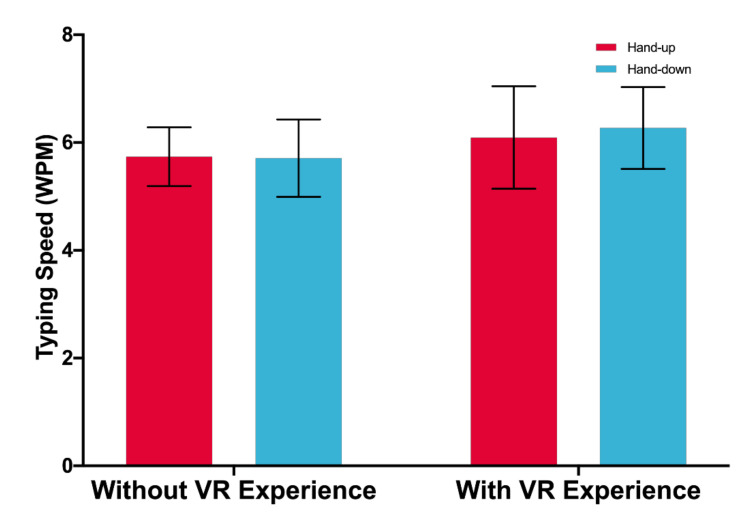
Words per minute. Error bars represent 95% CI.

No significant main effect of posture and VR experience on typing speed (WPM) is found: *F*(1,33) = 0.153, *p* = 0.669; *F*(1,33) = 0.969, *p* = 0.332, respectively. There is no significant interaction of posture and VR experience, *F*(1,33) = 0.291, *p* = 0.593. Figure 5 shows typing speed by posture and VR experience.

#### 4.1.2. Word Accuracy (WAcc)

**Figure 6:**
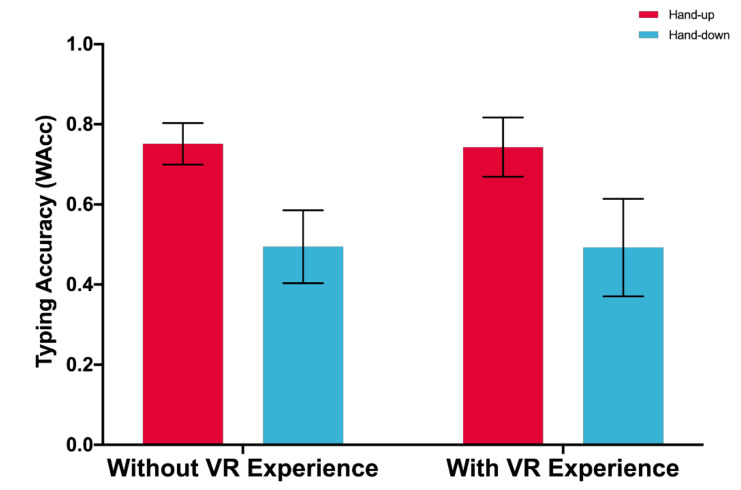
Word accuracy. Error bars represent 95% CI.

A significant main effect of posture on typing accuracy (WAcc) is found, with participants typing more accurately using hand-up posture (*M* = 0.747, *SD* = 0.123) than using hand-down posture (*M* = 0.493, *SD* = 0.207), *F*(1,33) = 57.460, *p* < 0.001. There is neither significant main effect of VR experience on typing accuracy, *F*(1,33) = 0.012, *p* = 0.915, nor interaction effect between posture and VR experience, *F*(1,33) = 0.008, *p* = 0.929 (see Figure 6).

#### 4.1.3. Learning Effect of Typing Accuracy

We conduct the paired T-test to compare each phase’s word accuracy in the practice phase and the experimental phase for the hand-up and the hand-down postures. There is no significant practice effect for typing accuracy between the practice and the experiment phase, *p* > 0.324.

### 4.2. Subjective Experience

#### 4.2.1. Perceived Usability

**Figure 7:**
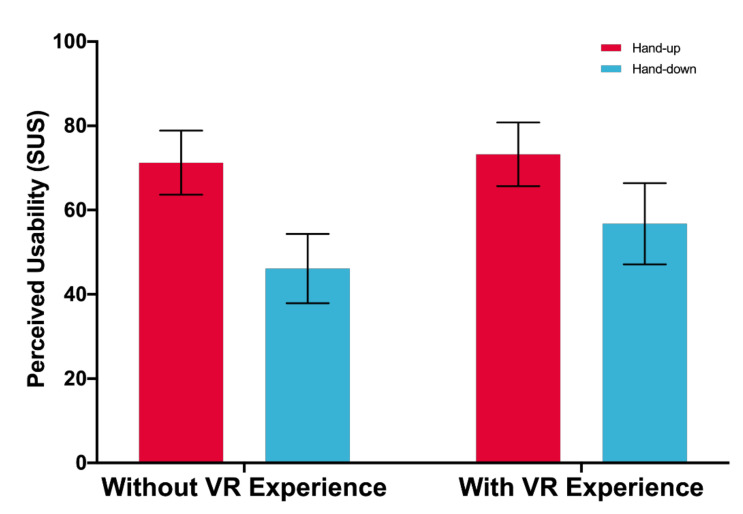
Perceived usability (100 = Highest). Error bars represent 95% CI.

A significant main effect of posture is found. Participants’ perceived usability when using the hand-up posture (*M* = 72.214, *SD* = 14.800) is higher than that when using the hand-down posture (*M* = 51.286, *SD* = 18.184), *F*(1,33) = 59.954, *p* < 0.001. There is no significant effect of VR experience, and no interaction effect between posture and VR experience: *F*(1,33) = 2.602, *p* = 0.116, *F* (1,33) = 1.707, *p* = 0.200, respectively (see Figure 7).

#### 4.2.2. User Experience

**Figure 8:**
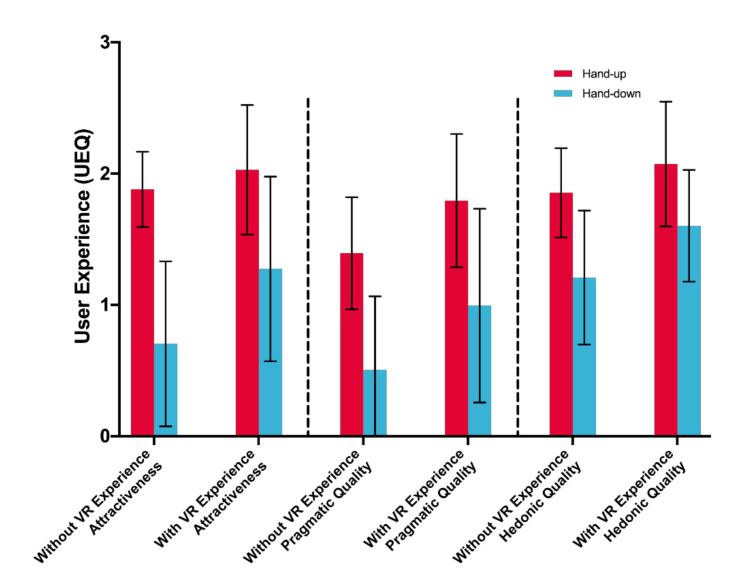
User Experience (3 = Highest). Error bars represent 95% CI.

The three user experience scores when using the hand-up posture are higher than those when using the hand-down posture. There are significant main effects of posture on three user experience dimensions: attractiveness, *F*(1,33) = 33.460, *p* < 0.001; pragmatic quality, *F*(1,33) = 26.586, *p* < 0.001; and hedonic quality, *F*(1,33) = 21.680, *p* < 0.001. No significant main effect of VR experience on user experience is found, *p* > 0.201, and no interaction effect of posture and VR experience is found, *p* > 0.201. Table 1 shows the average and standard deviation of all user experience scores, and Figure 8 shows the user experience scores.

#### 4.2.3. Motion Sickness

The main effects of posture and VR experience are not significant, *F*(1,33) = 0.932, *p* = 0.341, *F*(1,33) = 0.700, *p* = 0.793, respectively. There is no significant interaction effect between posture and VR experience, *F*(1,33) = 0.986, *p* = 0.328.

**Figure 9:**
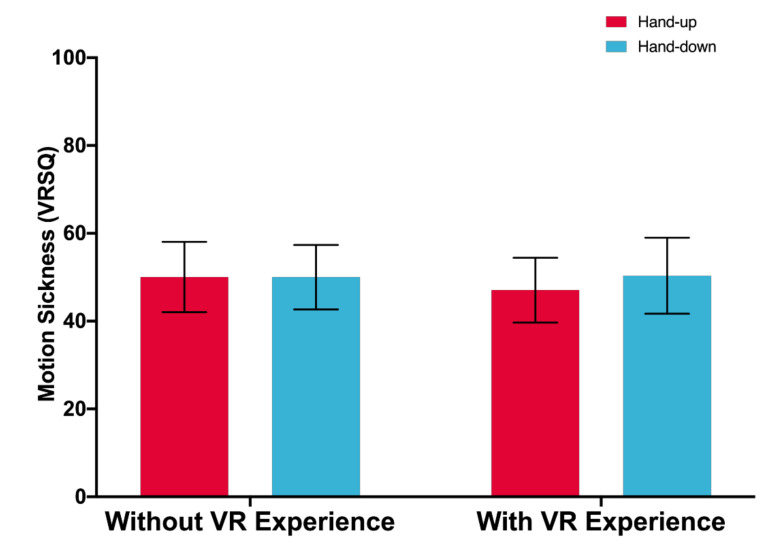
Motion sickness (100 = highest). Error bars represent 95% CI.

After two factors (O: Oculomotor, D: Disorientation) of VRSQ were analyzed, no significant effect of posture is found (O: *p* = 0.576, D: *p* = 0.138), and there is no significant effect of VR experience (O: *p* = 0.889, D: *p* = 0.466). No significant interaction effect is found (O: *p* = 0.167, D: *p* = 0.643). Figure 9 shows the motion sickness.

#### 4.2.4. Perceived Exertion

**Figure 10:**
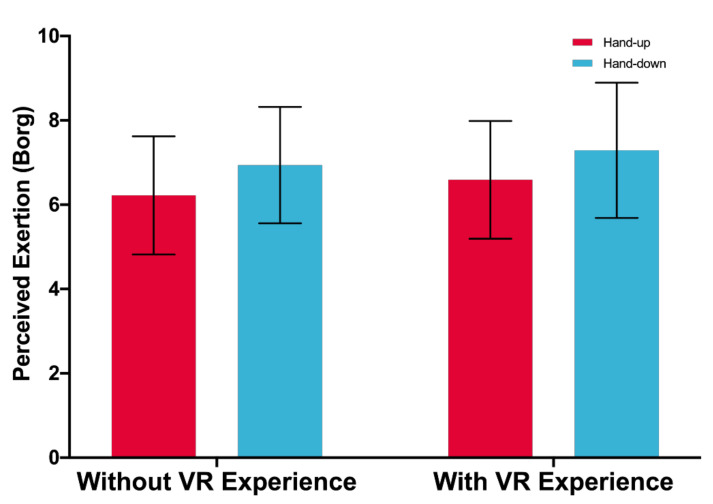
Perceived Exertion (10 = Highest). Error bars represent 95% CI.

No significant main effect of posture or VR experience on perceived exertion is found, *F*(1,33) = 3.893, *p* = 0.057, *F*(1,33) = 0.159, *p* = 0.692, respectively. There is no significant interaction effect between posture and VR experience, *F*(1,33) = 0.001, *p* = 0.982. Figure 10 shows the perceived exertion.

### 4.3. Task Workload

**Figure 11:**
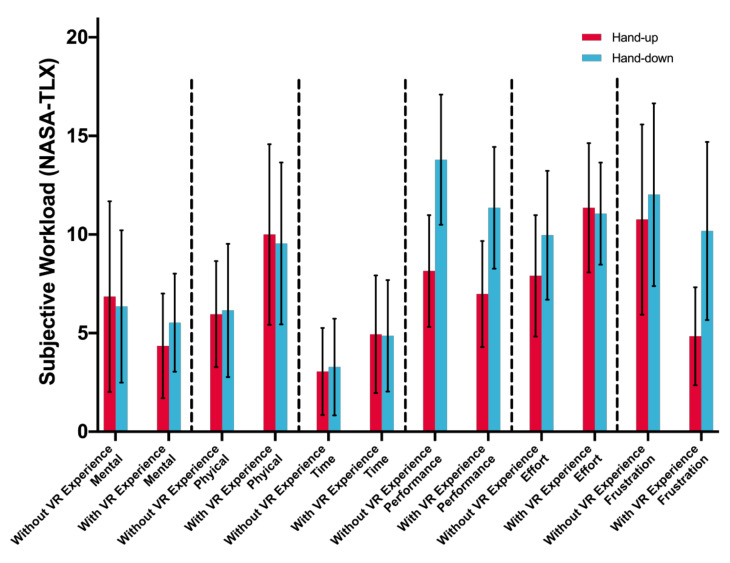
Task workload (six facets of NASA-TLX Scale) of participants when using different postures. Error bars represent 95% CI.

The overall task workload has a significant differences between two input postures, *F*(1,33) = 6.905, *p* = 0.013 (hand-up: *M* = 42.580, *SD* = 15.134; hand-down: *M* = 51.361, *SD* = 15.260). There is a significant main effect of posture on the performance facet. The task workload reported by participants when using hand-up posture (*M* = 7.580, *SD* = 5.424) is better than that when using hand-down posture (*M* = 12.609, *SD* = 6.366), *F*(1,33) = 14.437, *p* = 0.001. No significant interaction effect is found, *p* > 0.226, and for VR experience, there is no significant effect, *p* > 0.095. Table 2 and Figure 11 show the statistical analysis of task workload.

## 5. Discussion

In this study, we investigated the performance of mid-air text entry technology with different postures in a virtual environment. Correlations between independent variables (input posture and experience) and performance (typing performance, subjective experience, and task workload) were particularly studied. The findings revealed that the input posture has a great influence on input efficiency, accuracy, and user experience in the virtual environment. When participants used hand-up posture, they had better performance on accuracy (0.747 versus 0.493), perceived usability (72.214 versus 51.286), user experience (attractiveness: 1.952 versus 0.981, pragmatic quality: 1.588 versus 0.743, hedonic quality: 1.961 versus 1.400), and task workload (42.057 versus 51.886). Practical implications are drawn from these findings.

### 5.1. Spatial Cognition Consistency and Typing Performance

This study shows that input posture and the VR experience are two independent factors that have respective impacts on typing performance. As Figure 5 shows, neither input posture nor experience have a significant effect on typing speed, even though the typing speed of the participants with VR experience is numerically a little bit higher than that of the participants without VR experience (6.181 vs. 5.723). Figure 6 indicates that the input posture has a significant effect on typing accuracy. However, the effects of the VR experience are not significant.

Hand–eye coordination in virtual environments, which is an important embodiment of spatial cognitive ability, is a system in which the brain and body process actions and stimuli with eyes and transform them into actions and actions in real life. The muscle memory and spatial cognitive ability formed by users in daily life will directly affect the performance of text input. When users input text in virtual environments, they need to transfer the spatial cognitive rules from real life to virtual environments.

As mentioned above, for the hand-up posture, the upper limb is required to be perpendicular to the ground but parallel to the typing interface in the virtual environment. While for the hand-down posture, the upper limb is required to be parallel to the ground but perpendicular to the typing interface in VR. The spatial relationship between the input interface in the virtual environment and the participant’s upper limb and the whole body for the hand-up posture is closer to normal spatial interaction than that for the hand-down posture. It means that the hand-up posture has higher spatial cognition consistency and hand–eye coordination level than the hand-down posture in the mid-air text input.

When people control their hands to type on the virtual screen, their brains undergo extremely complex calculations about hand–eye coordination. It is one rule that uses visual guidance of the hand to strengthen hand–eye coordination to complete the precise control of the cursor on the screen by hands. First, participants need to make an eye movement toward the target position, then calculate the hand motor error in eye coordinates, and move the hand to the target position to compensate for the displacement deviation of the eyeball [51,52]. In this process, since the hand coordinates in the hand-up posture are parallel to the eye coordinates, hand-up posture can show faster and more accurate motor error compensation, allowing visual guidance of the hand to play a stronger role. It explains that the significant main effect of posture on typing accuracy, i.e., participants’ typing, is more accurate when using the hand-up posture.

Gorbet et al. [53] compared the performance of a hand-eye coordination task in different spatial planes and provided strong evidence for the above discussion. They asked the participants to complete two sets of tasks. In the standard task, participants were asked to slide along the vertical touchpad with their hands and move the cursor to the target on the vertical touchpad. In non-standard tasks, participants were asked to slide along the horizontal touchpad with their hand and move the cursor to the target on the vertical touchpad. Participants showed better intermediate accuracy and precision in the standard task. This shows that the performance in the hand–eye coordination task is indeed affected by the consistency between the hand coordinates and the eye coordinates. The coordinates consistency can improve users’ spatial cognitive consistency and achieve higher interaction accuracy.

These findings provide practical implications for VR devices with gesture cognition technologies for one hand interactions in a virtual environment, specifically for precise and long interactive applications. In previous studies, fatigue and vertigo were the focus of researchers (such as the gorilla arm effect), but this paper proves that spatial cognitive consistency has a great impact on interaction accuracy. In the future, when designers intend to use gesture cognition technologies for interaction, they should consider not only the fatigue caused by the gorilla arm effect, but also spatial cognitive consistency and hand–eye coordination to improve the accuracy of interaction.

### 5.2. Perceived Exertion, Task Workload, and User Experience

This study further reveals the effect of different hand postures on the mid-air input overall experience in the virtual environment. First, no significant main effect of posture or VR experience on perceived exertion was found, and there was no significant interaction effect between posture and VR experience. However, as Figure 10 shows, we found that both the hand-up and-down postures got a high value (6–8, between severe and highly severe) in the perceived exertion level of performing the input task. The results showed that it was relatively difficult to type in VR with mid-air text entry, compared to daily typing tasks on mobile devices.

Second, this study reveals the influence of different hand postures on the workload of the input task in a virtual environment. As shown in Table 2 and Figure 11, the overall task workload has a significant difference between the two input postures. Participants report a higher overall task workload when using hand-down posture than using hand-up posture. In the text input process, task workload is mainly reflected in two facets, i.e., performance and frustration. In a virtual environment, participants were dissatisfied with their performance and felt frustrated when using the hand-down posture to input. In ad hoc surveys at the end of the experiment, the participants generally reflected that because of the high error rate they perceived, they were easily frustrated and lost self-confidence in the process of the experiment, thus making it difficult to complete the task. This means that the hand-down posture is not an easy posture to use for text input. They cannot easily use this posture and obtain satisfactory performance. Comparatively, the hand-up posture is a posture that is easier to use.

Third, we identified a significant main effect of posture for perceived usability, attractiveness, pragmatic quality, and hedonic quality, but no significant main effect of VR experience for user experience, and no significant interaction effect between posture and VR experience. As Figure 7 and Figure 8 show, the perceived usability, attractiveness, pragmatic quality, and hedonic quality were higher when using the hand-up posture. This finding contributes new understandings about hand posture effects in a virtual environment to existing studies [54].

Participants got little motion sickness in this experiment. Nevertheless, there was no significant effect between posture and VR experience as well as their main effects on motion sickness, as shown in Figure 9. This finding confirms that although the sliding input in a virtual environment causes slight motion sickness, it has potential as an input mode in virtual environments in the future.

### 5.3. Summary

From the view of input efficiency and user experience of mid-air text input, this study supports that the hand-up posture, which has a better spatial cognition consistency, is a superior interaction posture than the hand-down posture. The previous research supports that the hand-up posture may cause higher fatigue than hand-down, so hand-down is more suitable for mid-air interaction [12]. Contrary to the previous research, in our study, the hand-up posture had better typing performance in virtual environments than the hand-down posture. We believe that fatigue is an important consideration when designing a mid-air interaction posture, but it should not be the only factor, especially for some tasks that require high interaction accuracy and high-level user experience.

Compared to a physical keyboard or a touchscreen keyboard, the input speed is not high (about 6 WPM), and the word accuracy is also relatively low (up to 74.7%). Possible reasons for these are explored, along with a suggestion of suitable scenarios for this text input method. Firstly, the keyboard layout we used needs to be optimized. The small spacing of the keyboard keys, which reduces the error tolerance rate of text input, leads users to control their hands more carefully and greatly reduces the input speed. Secondly, the current predictive model of the word-gesture keyboard was trained by the data of smartphone users. This model needs to be improved to adapt to the mid-air interaction and virtual environments. Third, users may need more time to master the using skills of the word-gesture keyboard, which is an unfamiliar input method for fresh users in virtual environments. Although long-term mid-air interaction usage faces the inevitable fatigue problem, there are some potential scenarios for the mid-air text input method in virtual environments. In games, presentations, virtual social, and other scenarios that do not require a large amount of text input, this input method is more advantageous, because it is more intuitive, natural, and immersive, but in scenarios that require a large amount of text input, such as office, writing, etc., designers should avoid using this input method.

## 6. Limitations

This study provides critical theoretical and practical implications, but there are a few experimental limitations. Limitations of this research include the small sample of participants and the different English reading and writing levels of participants involved in the study.

First, we only recruited 35 participants, including 18 participants without VR experience and 17 participants with VR experience. This sample size is relatively small, and we have not found any significant interaction between VR experience and input postures. We want to continue to explore the interaction between VR experience and input postures and recruit more participants for experiments.

Second, the 35 participants were recruited based on their experience in using VR devices, not on English level. The participants in the current study were all college students, so it would reduce the ecological validity to a certain extent.

## 7. Conclusions and Future Work

This article presents an empirical study to investigate the performance of gesture-based input for mid-air text entry with different input postures and VR experiences in a virtual environment. The study reveals that for the mid-air gesture-based text entry technology in the virtual environment, the VR experience has little effect on input performance on the whole, while the hand posture has a great influence on input performance, especially on typing performance, subjective experience, and task workload. The typing accuracy, perceived usability, and workload when using hand-up posture are obviously better than those when using hand-down posture, i.e., the typing accuracy and perceived usability level (<60) are too low to use. The study also finds that the perceived exertion of participants to complete text input with the mid-air gesture-based text entry technology is relatively high, so it is better to use short words or short sentences instead of long sentences in input tasks, which can reduce the time required for a single input. Furthermore, hand–eye coordination in the virtual environment is an important embodiment of spatial cognitive ability. For the hand-up posture, the spatial relationship between the input interface in the virtual environment and the participant’s upper limb and the whole body is closer to the normal spatial interaction when compared with the hand-down posture. Therefore, according to our study, we cautiously speculate that the hand-up posture has higher spatial cognition consistency and hand–eye coordination level in the mid-air text input when compared with the hand-down posture.

This study aims at investigating the performance of mid-air gesture-based text entry technology in a virtual environment. With the popularity of VR applications and the high-level of input precision requirement, there will be more scenarios where users need to change postures and positions in input tasks. In the future, we aim to design based on different kinds of sensors to record the physiological and psychological responses of text input in the virtual environment with the mid-air gesture-based text entry technology to master the overall impact of this technology on users. In addition, we also want to try more interactive postures and input methods to find a more suitable input method for virtual environments.

## Figures and Tables

**Table 1:** The average and standard deviation of all user experience scores on postures.

	Hand-Up	Hand-Down
Attractiveness ***	1.952 (0.778)	0.981 (1.328)
Pragmatic quality ***	1.588 (0.932)	0.743 (1.292)
Hedonic quality ***	1.961 (0.804)	1.400 (0.942)

***: *p* < 0.001, User experience score (3 = Highest).

**Table 2:** The average and standard deviation of all subjective workload scores on postures.

	Hand-Up	Hand-Down
Mental	5.638 (7.837)	5.952 (6.428)
Physical	7.924 (7.496)	7.800 (7.484)
Temporal	3.971 (5.157)	4.048 (5.197)
Performance **	7.580 (5.424)	14.722 (6.240)
Effort	9.581 (6.426)	10.495 (5.814)
Frustration	7.886 (8.182)	11.124 (8.971)
Overall *	42.581 (15.134)	51.362 (15.260)

* *p* < 0.05, **p < 0.01.
